# Chemodectoma Arising From the Abdominal Aorta in a 9‐Year‐Old Female Dog: Clinical, Histopathological and Immunohistochemstric Study

**DOI:** 10.1002/vms3.71321

**Published:** 2026-11-05

**Authors:** Seyed Amirreza Boskabadi, Khosro Safari Nikroo, Ali Mirshahi, Hossein Nourani, Seyed Sajad Hadiyan

**Affiliations:** ^1^ Faculty of Veterinary Medicine Ferdowsi University of Mashhad Mashhad Iran; ^2^ Department of Clinical Sciences, Faculty of Veterinary Medicine Ferdowsi University of Mashhad Mashhad Iran; ^3^ Department of Pathobiology, Faculty of Veterinary Medicine Ferdowsi University of Mashhad Mashhad Iran

**Keywords:** extra‐cardiac chemodectoma, histopathology, immunohistochemistry, neuroendocrine tumour, synaptophysin, veterinary oncology

## Abstract

**Background:**

Chemodectomas are uncommon, typically benign neoplasms derived from paraganglionic chemoreceptor tissues, most frequently arising in the heart base or carotid bodies of brachycephalic dogs. This report documents a detailed pathological description of a chemodectoma originating from the abdominal aorta in a non‐human species, representing, to our knowledge, the first case specifically characterized by its origin at the aortic trifurcation, thereby expanding the recognized anatomical spectrum of this tumour entity.

**Case Presentation:**

A 9‐year‐old spayed female terrier was presented with persistent panting, recent‐onset obesity following previous cachexia, and a complex clinical history including polyuria/polydipsia and pyoderma. Haematological and biochemical analyses revealed a regenerative anaemia, leucocytosis, and biochemical indicators suggestive of cholestasis and potential endocrinopathy. Advanced imaging identified a solitary, well‐demarcated, highly vascular abdominal mass closely associated with the aortic trifurcation. Given the tumour's inaccessibility and pronounced vascularity, cytological confirmation via fine needle aspiration was precluded due to haemorrhagic risk, necessitating exploratory laparotomy.

**Management and Outcome:**

Intraoperatively, the mass was unresectable because of its anatomical relationships and the prohibitive risk of catastrophic haemorrhage. Humane euthanasia was subsequently elected. Histopathological evaluation demonstrated a lobulated neoplasm composed of pleomorphic neuroendocrine cells, with low mitotic activity and an absence of necrosis or haemorrhage. Immunohistochemistry confirmed strong, diffuse synaptophysin reactivity and focal neuron‐specific enolase positivity, while chromogranin A and S100 protein were negative, underscoring immunophenotypic variability.

**Conclusion:**

This case illustrates the diagnostic complexity and therapeutic limitations posed by extra‐cardiac chemodectomas in veterinary species, especially when located in unconventional anatomical sites. Limitations included the unavailability of computed tomography and lack of postoperative follow‐up. This report expands the recognized clinico‐pathological spectrum of chemodectomas and highlights the necessity for multidisciplinary diagnostic strategies and further research aimed at defining optimal management protocols for such rare and challenging presentations.

## Graphical abstract

A 9‐year‐old spayed female dog presented with a solitary, well‐demarcated, intensely vascular abdominal mass closely associated with the aortic trifurcation on advanced imaging. Exploratory laparotomy confirmed that the lesion was unresectable because of its anatomical relationships and the risk of catastrophic haemorrhage. Histopathology and immunohistochemistry confirmed a chemodectoma with strong synaptophysin reactivity, representing the first reported canine case originating at this location.

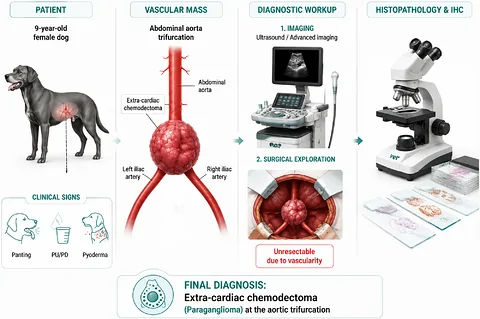

**Abbreviations**
ALP: alkaline phosphatase
CBC: complete blood count
CD: chemodectoma
CT: computed tomography
FBS: fasting blood sugar
FNA: fine needle aspiration
GGT: gamma‐glutamyl transferase
NSE: neuron‐specific enolase
PU/PD: polyuria/polydipsia
US: ultrasonography
VD: ventrodorsal

## 1 Introduction

Chemodectomas (CDs) are soft, nodular, highly vascular, encapsulated, homogeneous masses arising from chemoreceptors, mainly located around the carotid bifurcation, the aortic root, the main pulmonary arteries, and the jugular vein (Bussadori 2015). Although classified as cardiac tumours, chemoreceptor tissues are distributed throughout the body and play a critical role in regulating respiration and circulation. In animals, these neoplasms predominantly develop in the aortic bodies, whereas carotid body tumours are more prevalent in humans. Older brachycephalic dog breeds, such as English and French Bulldogs, Boxers, and Boston Terriers, are at a higher risk for developing CDs (Gómez Ochoa et al. 2021). However, cases have been documented in cats, cattle and horses (Khodakaram‐Tafti et al. 2011; Saunders et al. 2021; Knottenbelt et al. 2015). Physiologically, decrease in arterial oxygen, hyperthermia, acidosis and hypercapnia could trigger the aortic bodies. In humans, chronic hypoxia and genetic predispositions have been implicated in tumorigenesis (Coto et al. 2021). Most CDs are typically benign, but they can compress adjacent structures, causing mass‐effect symptoms (Gómez Ochoa et al. 2021; Verghis et al. 2023) including ascites, pleural and pericardial effusion, exercise intolerance, cough, lethargy, anorexia, inappetence, collapse, and arrhythmias. These tumours are incidentally discovered during echocardiography, X‐rays, US examinations or necropsy (Gómez Ochoa et al. 2021); however, diagnosis may sometimes be based on signalment, history and physical examination. In cases where the mass is anatomically accessible, a definitive diagnosis can be obtained through fine needle aspirates (FNA) or tissue biopsy, although this is usually challenging due to the anatomical location and vascularity (López‐Arcas et al. 2022; Kemppainen 2023). A variety of therapeutic modalities have been suggested for CD, including surgical excision, radiotherapy, chemotherapy and palliative interventions (Gómez Ochoa et al. 2021). The optimal treatment depends on tumour location, resectability, patient status and owner preferences. While surgery is preferred when feasible, many cases require alternative or adjunctive therapies due to various constraints.

While CDs originating from the aorta have been sporadically reported in non‐canine species, this study presents the first comprehensive pathological and immunohistochemical characterization of a histologically benign CD arising specifically from the abdominal aorta trifurcation in an animal, thereby expanding the recognized anatomical and biological spectrum of this neoplasm.

## 2 Case Presentation

A 9‐year‐old spayed female terrier presented with persistent panting and recent obesity following a previous episode of cachexia. The dog had a history of fungal infection, untreated polyuria/polydipsia (PU/PD) and pyoderma. Upon presentation, a thorough examination was performed, including a complete blood count (CBC) and a serum biochemical analysis. The CBC revealed anaemia with polychromasia, suggesting a regenerative response. Additionally, leucocytosis with an increased band cell count and toxic changes in the white blood cells were observed, which were highly suggestive of an acute inflammatory process driving accelerated granulopoiesis. The biochemical panel showed elevated fasting blood sugar (FBS), cholesterol, alkaline phosphatase (ALP), and gamma‐glutamyl transferase (GGT). The elevated ALP and GGT were indicative of possible cholestasis. Increased fasting blood glucose and cholesterol raised concerns for diabetes mellitus. Concurrently, the dog exhibited hypocalcaemia, hyponatremia, hypochloraemia, and hypokalaemia, though other values were within normal limits.

### 2.1 Diagnostic Imaging and Surgical Challenges

A transabdominal ultrasound revealed a solid, solitary, well‐defined, hyperechoic, heterogenous, and highly vascular mass, approximately 12 × 12 cm in diameter, located near the abdominal aortic trifurcation. Doppler imaging confirmed increased blood flow to the mass, consistent with its vascular nature (Figure 1). In the right lateral and ventrodorsal (VD) radiographs of the abdominal cavity, a soft tissue solid mass was seen between the cranial aspect of L6 and the caudal aspect of L1. Serosal detail and margin were obliterated in cranial and mid‐abdomen which may have been due to peritoneal effusion or a soft tissue mass. Cardiomegaly and bronchointerstitial pattern in all of lung lobes were shown in thoracic radiographs; however, no sign of metastatic lesions was detected in thoracic cavity. Given the tumour's vascularity and location, FNA was not performed due to the high risk of bleeding. Instead, surgical exploration was chosen to evaluate the feasibility of resecting the mass. During surgery, it was determined that the mass was unresectable due to its extensive vascularization and proximity to major blood vessels (Figure 2). The size and location of the tumour posed a significant risk of severe haemorrhage, and the likelihood of surgical complications was deemed too high. After careful consideration of the poor prognosis, euthanasia was performed during the procedure to prevent further suffering. After euthanasia, several samples of the neoplastic mass were collected.

**FIGURE 1:**
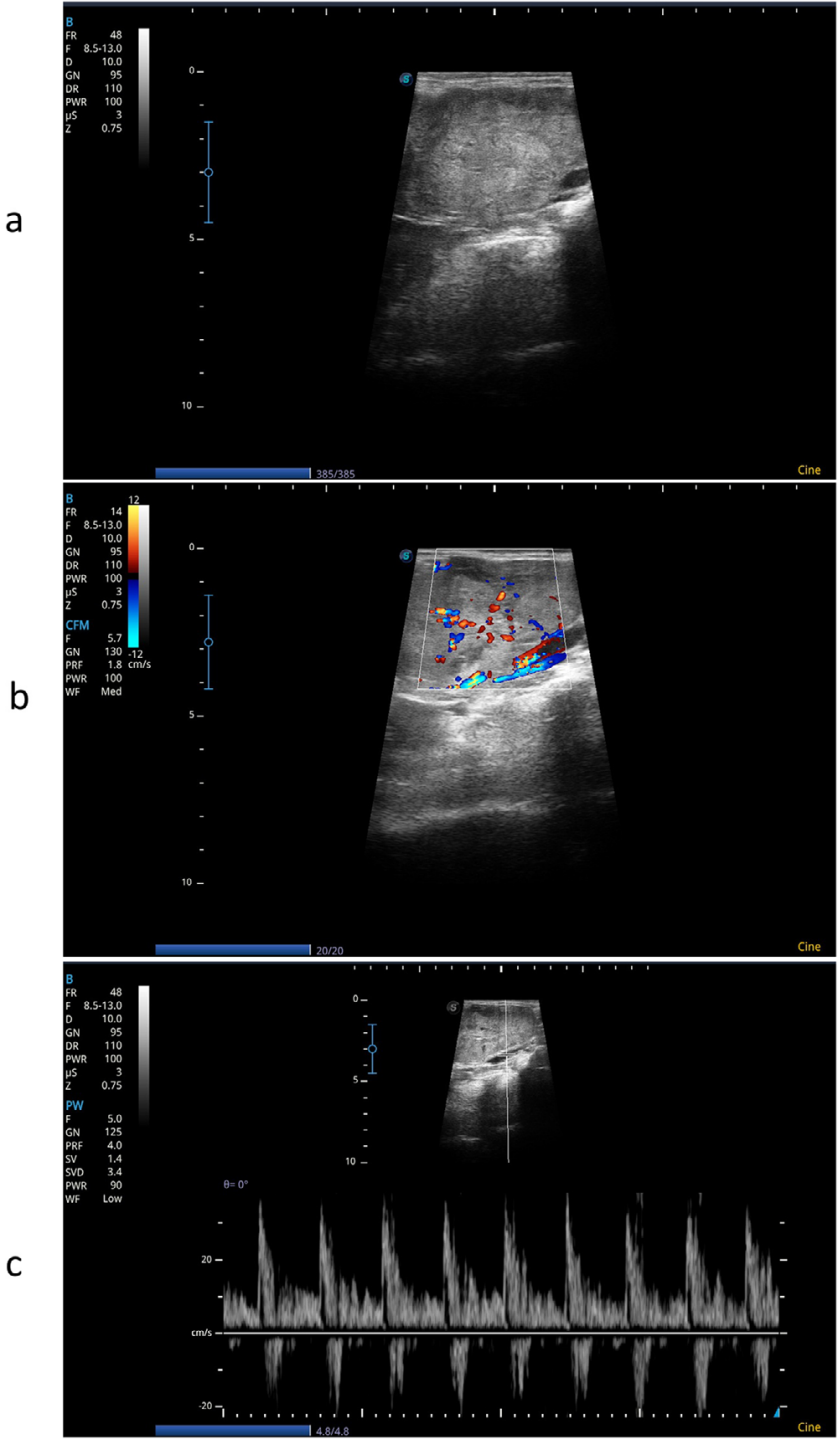
Ultrasound imaging. (a) Sagittal, transabdominal ultrasound image showing a well‐defined, solid, heterogeneous and hyperechoic mass, located near the aortic bifurcation. (b) Colour Doppler ultrasound image demonstrating marked internal vascularity within the abdominal mass, confirming its hypervascular nature. Multiple colour flow signals are visualized throughout the lesion, consistent with increased blood supply. (c) Pulsed‐wave Doppler ultrasound of the abdominal aorta showing arterial flow within the lesion, indicating that the mass originates from or is closely associated with the aortic wall.

**FIGURE 2:**
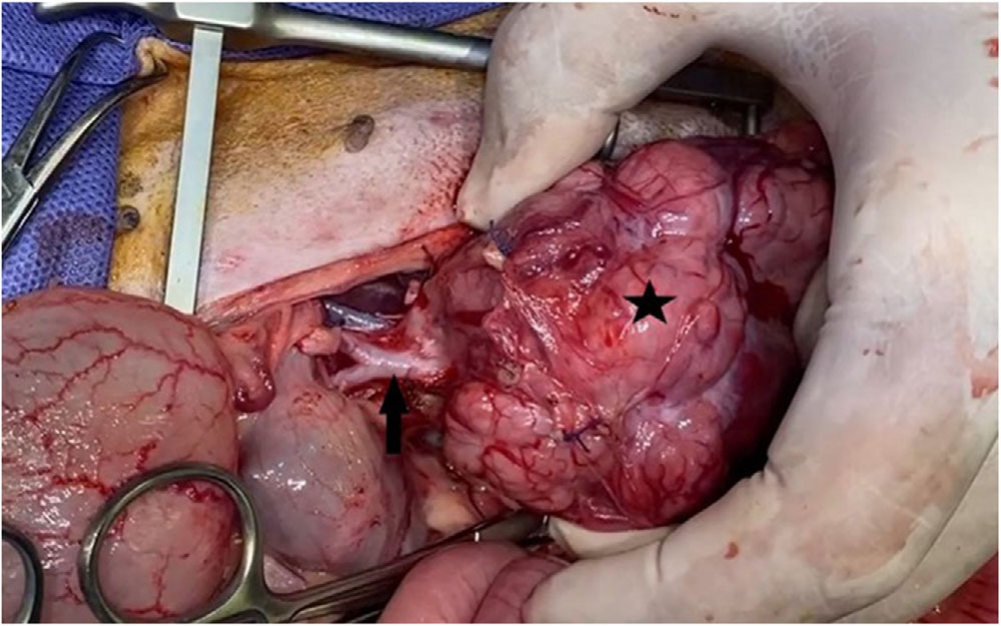
Perioperative view of tumour. Note multilobular neoplastic mass (asterisk) adjacent to abdominal aorta (arrow).

**FIGURE 3:**
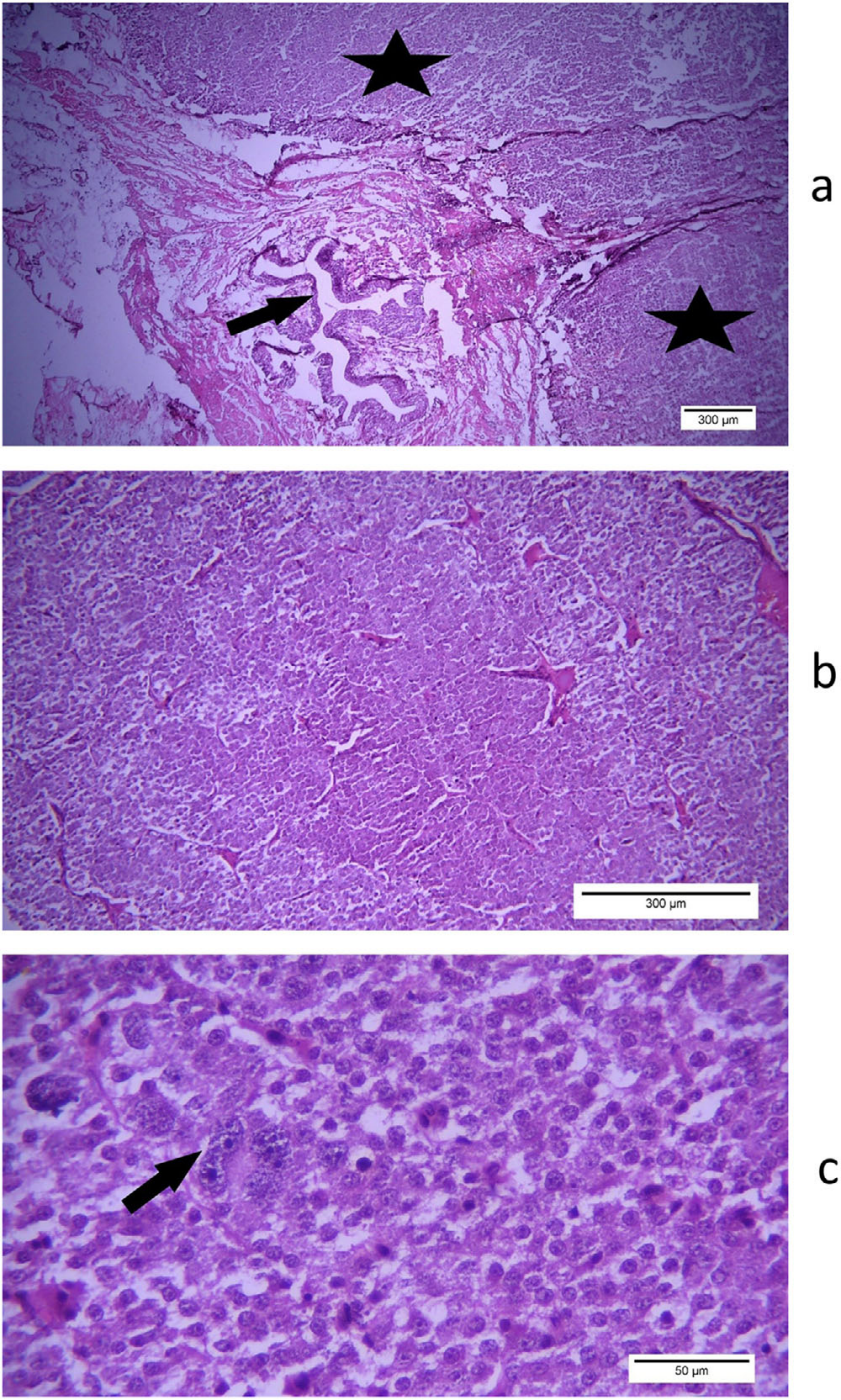
(a) Histopathology: neoplastic area (asterisks) are seen adjacent to the ureter section (arrow) (Haematoxylin and Eosin, bar = 300 µm). (b) Histopathology: neoplastic cells clusters are separated by delicate connective tissue with capillaries (Haematoxylin and Eosin, bar = 300 µm). (c) Histopathology: some neoplastic cells have cytomegaly and karyomegaly (arrow) (Haematoxylin and Eosin, bar = 50 µm).

**FIGURE 4:**
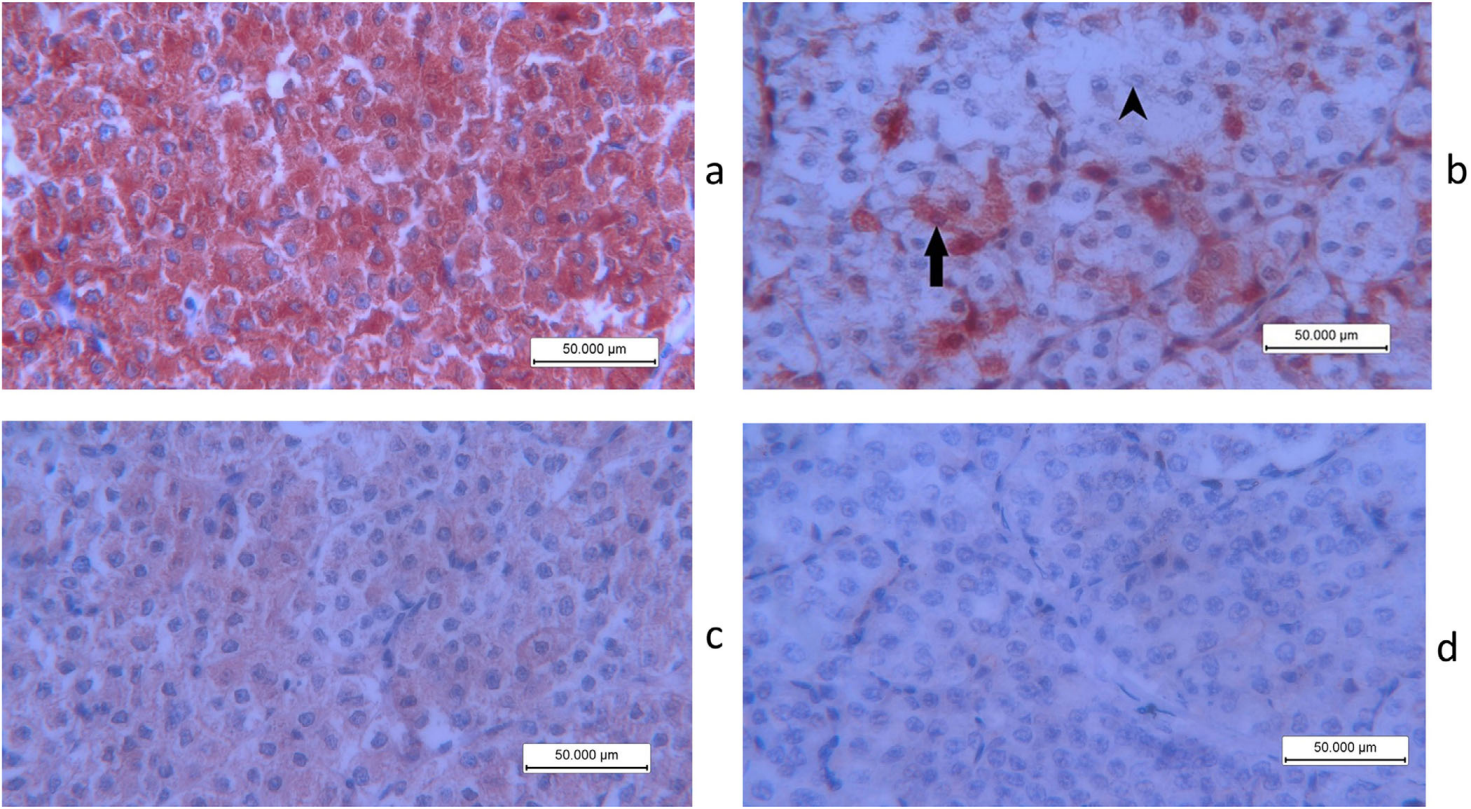
(a) Immunohistochemistry: diffuse and intensive positivity for synaptophysin (AEC [3‐amino‐9‐ethylcarbazole] as the chromogen, immunohistochemistry, bar = 50 µm). (b) Immunohistochemistry: the neoplastic cells reaction result for neuron specific enolase was different including positive (arrow) and negative (arrowhead) (AEC [3‐amino‐9‐ethylcarbazole] as the chromogen, immunohistochemistry, bar = 50 µm). (c) Immunohistochemistry: diffuse negative immunohistochemistry reaction for S100 (AEC [3‐amino‐9‐ethylcarbazole] as the chromogen, immunohistochemistry, bar = 50 µm). (d) Immunohistochemistry: diffuse negative immunohistochemistry reaction for chromogranin A (AEC [3‐amino‐9‐ethylcarbazole] as the chromogen, immunohistochemistry, bar = 50 µm).

### 2.2 Histopathology and Immunohistochemical Staining

Tissue samples from the tumour mass were fixed in 10% natural buffered formalin, routinely processed, embedded in paraffin wax and sections of 5 µm thickness were stained with haematoxylin and eosin. In addition, consecutive sections to those used for the histopathological examination were subjected to immunohistochemistry using the avidin–biotin complex method with AEC (3‐amino‐9‐ethylcarbazole) employed as the chromogen for visualization. Antigen retrieval was performed using a Tris‐based solution. Subsequently, the sections were incubated with primary antibody against synaptophysin, neuron‐specific enolase (NSE), chromogranin A and s100 protein. All primary antibodies were from Vitro (Master Diagnostica), Seville, Spain. Positive controls used in this study for the immunohistochemistry tests were pancreas for synaptophysin, NSE and chromogranin A and melanoma for s100 protein. Microscopic examination revealed the tumour subdivided into lobules by branching trabeculae of connective tissue, containing collagen and small capillaries. Some neoplastic lobules were observed adjacent to the ureter section (Figure 3a). Pleomorphic neoplastic cells were arranged as clusters and round to polyhedral in shape with variable amount of lightly eosinophilic and granular cytoplasm around the nucleus. The nuclei of tumour cells were round to oval or irregular with prominent nucleoli and were placed centrally or marginally in the cells (Figure 3b,c). The mitotic activity was low with less than one mitotic figure per 10 × 40 fields. There were no areas of haemorrhage or necrosis in the tumour tissue.

In immunohistochemistry, diffuse and intensive immunoreactivity was seen for synaptophysin (Figure 4a). The immunohistochemistry result for neuron specific enolase was variable, and about 30% of neoplastic cells stained positively and strongly (Figure 4b). Diffuse negative immunohistochemistry reaction was observed for S100 protein and chromogranin A (Figure 4c,d). On the basis of the clinicopathological and immunohistochemical findings, the tumour was diagnosed as abdominal aortic body CD.

## 3 Discussion

This case report documents a unique, histologically benign CD originating from the abdominal aorta trifurcation, expanding the known anatomical distribution of these tumours. Unlike the typical locations such as heart base (Arantes et al. 2024) or other thoracic locations (Martinez et al. 2022), this tumour's rare presentation highlights new challenges in diagnosing and managing CDs in nontraditional sites. Furthermore, this case contributes to the limited but growing body of literature on CDs occurring in non‐canine species, suggesting that the anatomical spectrum for these neoplasms is broader than traditionally assumed. Its vascular nature and proximity to major blood vessels made surgical treatment impossible, emphasizing the need for further research into alternative therapies for such cases. While abdominal chemoreceptors have been identified anatomically, their role in tumour development remains poorly understood. This observation introduces the possibility of ectopic chemoreceptor tissue playing a role in tumour formation in unusual anatomical locations. A review of prior cases from the literature allows for a comparative analysis of CDs, particularly with respect to their anatomical location, diagnostic strategies and clinical outcomes.

CDs in animal species are most commonly reported in the heart base (Johannsen et al. 2025; Feitosa et al. 2021), carotid bodies (Souza et al. 2022), cranial vena cava (Martinez et al. 2022) and also abdominal metastatic from aortic origin (Hansen et al. 2016). These locations correlate with the concentration of chemoreceptor tissue, which plays a role in respiratory and circulatory regulation. Deregulation of chemoreceptors, which detect changes in pH and partial oxygen and carbon dioxide pressures, can result in hyperpnea and dyspnoea as previously mentioned studies (Martinez et al. 2022; Feitosa et al. 2021). The abdominal aorta, being anatomically distant from the typical sites of tumour occurrence, is less likely to present with the standard circulatory and respiratory clinical signs associated with such neoplasms.

In our study, abdominal radiography and ultrasound served as the primary diagnostic modalities for identifying the neoplastic mass. In contrast, previous reports have predominantly utilized chest X‐ray, echocardiography, computed tomography (CT) and post‐mortem examinations to detect CDs in traditional locations. This difference in diagnostic approach is likely attributable to the atypical and uncommon site of the CD in our case, which may not have been readily detectable using the standard diagnostic techniques employed for tumours at the heart base or within the thoracic cavity (Martinez et al. 2022; Souza et al. 2022; Crawford‐Jennings et al. 2025; Adetunji et al. 2023).

Typical clinical signs associated with CDs includes cough, dyspnoea, pleural effusion, cyanosis and dysphagia (Saunders et al. 2021; Arantes et al. 2024; Adetunji et al. 2023; Leite et al. 2024), which are generally attributed to the mass effect and compression of adjacent thoracic structures. However, unlike previous reports, most clinical signs in this case were nonspecific, due to the absence of cardiopulmonary symptoms typically seen with heart base tumours. The lack of cardiopulmonary mass sets this case apart, highlighting the variability in clinical presentation based on tumour location.

In comparison to previously reported cases of CD or extra‐adrenal paraganglioma, the present study demonstrated several important histopathological and immunohistochemical similarities and differences. Consistent with earlier findings (Saunders et al. 2021; Adetunji et al. 2023; Stylianaki et al. 2024), our tumour displayed a classic lobulated architecture with neoplastic polygonal to round cells arranged in clusters and separated by fibrovascular stroma. Like most published cases (Saunders et al. 2021; Adetunji et al. 2023; Aceino et al. 2020), mitotic activity in our tumour was low, and there was an absence of necrosis or haemorrhage, aligning with the benign spectrum of the disease reported in several studies. However, malignancy is also reported in a feline patient (Hansen et al. 2016). Immunohistochemically, strong and diffuse synaptophysin positivity and focal NSE expression in our case echo the neuroendocrine nature of CDs described in the literature (Saunders et al. 2021; Hansen et al. 2016; Adetunji et al. 2023; Stylianaki et al. 2024; Aceino et al. 2020). However, unlike many previous reports, our tumour exhibited a complete lack of immunoreactivity for chromogranin A (Hansen et al. 2016) and S100 protein, whereas most comparable cases demonstrate at least partial positivity for one or both markers (Saunders et al. 2021; Adetunji et al. 2023; Stylianaki et al. 2024). Crucially, this chromogranin A‐negative profile has been reported in overtly malignant aortic body tumours, which also showed abdominal metastases. This underscores that the absence of chromogranin A does not exclude malignant potential in atypical sites, validating the need for thorough staging in our case (Hansen et al. 2016). The absence of these markers in our sample highlights known immunophenotypic variability in aortic body tumours and underscores the importance of using a panel of neuroendocrine markers in diagnosis. Overall, our findings support the characteristic histology of CD but broaden the recognized spectrum of immunohistochemical expression for this tumour type.

Management strategies for CD in animals, as documented in the literature, encompass a broad spectrum of approaches, including chemotherapy, surgical excision, radiation therapy, palliative interventions and—when necessary—euthanasia. The choice of therapy is largely determined by tumour resectability, anatomical location and the patient's overall clinical status. For instance, toceranib, a tyrosine kinase inhibitor, has been utilized as a chemotherapeutic agent in non‐resectable cases (Coto et al. 2021). Also, surgical excision via thoracotomy has been reported as the preferred treatment in a feline case (Martinez et al. 2022). Radiation therapy, both as a standalone (Gómez Ochoa et al. 2021; Magestro et al. 2018) and in combination with surgical resection (López‐Arcas et al. 2022), has also been documented as an effective treatment modality. Other studies describe palliative approaches such as transvalvular pulmonary artery stent angioplasty (Winter et al. 2023) and the administration of Theranekron to alleviate clinical symptoms (Szalus et al. 2021). Nevertheless, as noted in previous studies, there are cases in which these interventions do not yield a favourable response (Saunders et al. 2021; Aceino et al. 2020), making euthanasia the most appropriate option, a finding consistent with the outcome observed in the present study.

In conclusion, this report documents a rare presentation of abdominal aortic CD in a veterinary species, contributing to the limited literature on extra‐cardiac CDs. The case underscores important diagnostic and management challenges associated with this tumour type, particularly in atypical anatomical locations. However, the interpretation of the findings is limited by the absence of advanced imaging modalities such as CT scan, the inability to obtain a preoperative tissue diagnosis due to the risk of haemorrhage, and the lack of long‐term follow‐up as a result of intraoperative euthanasia. As this account pertains to a single case, broader conclusions cannot be drawn, and further studies are warranted to better inform the diagnosis and management of similar cases in the future.

## Author Contributions

**Khosro Safari Nikroo**: writing – original draft, writing – review and editing, investigation. **Seyed Sajad Hadiyan**: writing – original draft. **Hossein Nourani**: writing – original draft, formal analysis, investigation. **Ali Mirshahi**: writing – review and editing, formal analysis, investigation. **Seyed Amirreza Boskabadi**: data curation, writing – original draft.

## Funding

The authors have nothing to report.

## Ethics Statement

Ethics approval was not required for this case report as per institutional guidelines.

## Conflicts of Interest

The authors no conflicts of interest.

## Data Availability

The original contributions presented in the study are included in the article. Further inquiries can be directed to the corresponding author.
